# Leveraging organoid models to understand mechanisms of viral infections and immunity in bats

**DOI:** 10.1242/dmm.052737

**Published:** 2026-05-13

**Authors:** Shelby R. Madden, Agnieszka Rynda-Apple, Diane Bimczok

**Affiliations:** Department of Microbiology and Cell Biology, College of Agriculture, Montana State University, Bozeman, MT 59718, USA

**Keywords:** Bat, Chiroptera, Organoids, *In vitro* model, Infection, Antiviral response

## Abstract

Bats are important reservoir hosts for zoonotic viruses owing to their unusual ability to avoid development of clinical disease and pathological lesions upon viral infection. Research efforts to understand the unique responses of bats to viral infection have been limited by the vast number of bat species and the lack of accessible experimental model systems. Over the past 5 years, organoid models, which are long-term cultures of primary cells, have been developed from gastrointestinal, respiratory and kidney tissues of multiple fruit-eating and insectivorous bats*.* Compared with human organoids, bat organoids showed increased expression of type I and type III interferon genes, other antiviral genes and complement genes at baseline and in response to viral stimulation. Bat organoids generally showed strong upregulation of interferon genes and interferon-stimulated genes in response to viruses. Conversely, the ability of bat organoids to support viral replication was dependent on the virus, tissue type and bat species. Overall, the recent progress in the field demonstrates the potential of organoids to serve as relevant models for exploring species- and tissue-specific responses to viruses in bats.

## Teaser

**Summary:** Bat organoids are a newly emerging technology that is effective for studying infection dynamics and immunological responses to viral infection *in vitro* in relevant primary cells.

## Introduction

Bats are interesting and unique creatures. They belong to the second largest mammalian order (Chiroptera), with over 1400 species, and are thought to be among the most abundant vertebrates on Earth (Banerjee et al., 2020; Calderon et al., 2016; Hoar et al., 1998; Moratelli and Calisher, 2015; Szentivanyi et al., 2024). The unique ability of bats for powered flight, which uses muscular power for active aerial locomotion, is a special adaptation that distinguishes bats from all other mammals and is associated with unique metabolic functions (Calisher et al., 2006). Bats also display an elevated life expectancy relative to their body size (Wilkinson and Adams, 2019), with some bat species, such as Brandt's bat (*Myotis brandtii*) living up to 40 years (Podlutsky et al., 2005). More details of bat adaptations and biology are reviewed elsewhere (Sadier et al., 2020). Research from the past 20 years has revealed that bats have incredibly diverse viromes, which include zoonotic viruses that can cause disease in humans (Anthony et al., 2017; Banerjee et al., 2018; McElhinney et al., 2018; Munster et al., 2016; Ruiz-Aravena et al., 2022; Schountz et al., 2017). Zoonotic viruses isolated from bats include lyssaviruses such as rabies virus (McElhinney et al., 2018), coronaviruses (Balboni et al., 2011; Lau et al., 2019; Zhou et al., 2020), filoviruses such as Marburg virus (MARV) (Towner et al., 2009), and henipaviruses such as Hendra virus (Becker et al., 2023; Madera et al., 2022). Although most of these pathogens are dangerous to humans, bats may carry these viruses without exhibiting signs of infection (Calisher et al., 2006; Hoar et al., 1998). For example, Hendra virus infects bats (Becker et al., 2023), is shed by them (Paez et al., 2017; Peel et al., 2019) and may cause histopathological changes (Williamson et al., 2000) but does not cause clinical disease (Peel et al., 2022). Similarly, bats can carry Ebola virus without developing the hemorrhagic disease typically seen in humans (Jones et al., 2015). A notable exception to this observation are lyssaviruses, which can cause neurological signs and death in multiple bat species, including Mexican free-tailed bats (*Tadarida brasiliensis*) (Baer and Bales, 1967; Burns et al., 1958), serotine bats (*Eptesicus serotinus*) (Freuling et al., 2009), Daubenton's bats (*Myotis daubentonii*) (Johnson et al., 2008), straw-colored fruit bats (*Eidolon helvum*) (Begeman et al., 2020) and flying foxes (*Pteropus* spp.) (Barrett et al., 2020; Field et al., 1999). Notably, only a small number of zoonotic viruses are known to be transmitted directly from bats to humans. For example, lyssaviruses can be spread through bites or scratches from insectivorous bats, fruit bats, vampire bats (Kuzmin et al., 2011) and nectar-feeding bats, including flying foxes (*Pteropus* spp.) (Lateef, 2025). MARV is thought to be transmitted to humans through direct exposure to fluids from Egyptian fruit bats (*Rousettus aegyptiacus*) in African caves during the bats' birthing season (Amman et al., 2012). Other bat viruses may require an intermediate mammalian host, such as Dromedary camels for Middle East respiratory syndrome β-coronavirus (MERS-CoV) and horses for Hendra virus (Lau et al., 2017; Playford et al., 2010). Nipah virus is believed to be transmitted from bats to humans through contaminated food sources shared by bats and humans and through close contact with infected pigs (Chua et al., 2002; Gurley et al., 2017).

The identification of Horseshoe bats (*Rhinolophus* spp.) as a probable source of severe acute respiratory syndrome coronavirus 1/2 (SARS-CoV-1/2)-related coronaviruses has led to increased interest in the investigation of bat-borne pathogens with potential for cross-species transmission (Andersen et al., 2020; Li et al., 2005; Zhou et al., 2021, 2020). An accumulating body of evidence identifying bats as potential hosts for viruses that affect humans made understanding how bats evade clinical disease upon viral infection, while still transmitting viruses to human and intermediate hosts, a high priority. Biologically relevant methods and tools for studying viral transmission and immune responses in bats are essential for these investigations. In this Review, therefore, we will discuss traditional experimental models to study infection and immunity in bats, provide an overview of organoids as improved tissue culture models, explain recent research in the development of organoid cultures from bat species, and highlight new research findings from bat organoids as models of viral infection and antiviral immunity.

## Experimental systems to study bat cell function

### Traditional tissue culture systems in bats

Bat immunological research has previously involved primary and immortalized cell lines, *in vivo* experiments with laboratory-bred or wild-caught bat colonies, and analysis of field samples collected from wild bats (Ahn et al., 2023; Lau et al., 2019). Investigators have generated immortalized cell lines representing different organ systems, mostly kidney and respiratory tract, from Old and New World bats, including black flying foxes (*Pteropus alecto*), greater mouse-eared bats (*Myotis myotis*) and Jamaican fruit bats (*Artibeus jamaicensis*) (Banerjee et al., 2018). A comprehensive overview of bat cell lines is included in a review by Banerjee et al. (2018). Although these culture systems have been incredibly useful for studying bat viruses in species-specific cells, they have limitations, such as the short lifespan of primary cell lines or the accumulation of chromosomal aberrations in immortalized cells (Banerjee et al., 2018), and a lack of representation of multiple cell types present *in vivo* (Tang et al., 2022). Therefore, there has been a growing need for more sophisticated systems that maintain the structural organization and cellular complexity of bat tissues (Gunther et al., 2022; Tang et al., 2022).

### Organoids as advanced tissue culture systems

Organoids are self-arranging three-dimensional (3D) structures derived from stem cells that mimic the composition and organization of complex tissues and organs (Tang et al., 2022). Since the initial description of intestinal organoids as a tissue culture tool by Sato et al. (2009), the organoid field has expanded exponentially (Corro et al., 2020; Tang et al., 2022). Organoids can be derived from induced pluripotent stem cells, embryonic stem cells or adult somatic stem cells (Corro et al., 2020; Kawasaki et al., 2022; Tang et al., 2022). Importantly, as organoids are derived from untransformed primary cells, they maintain physiological characteristics and functions of their tissues of origin. Another important advantage of organoids is their ability to maintain genetic signatures of their host tissues over many passages (Corro et al., 2020; Kawasaki et al., 2022; Tang et al., 2022). The growth factors required to maintain the self-renewing stem cell population, which allows long-term culture of organoids, are highly conserved among species; thus, similar media can be used to derive and maintain organoids from a wide variety of animal tissues (Powell and Behnke, 2017).

Human organoids can be developed from patient tissues and have been used successfully in numerous studies to investigate infectious diseases (Bartfeld et al., 2015; Blutt et al., 2018), carcinogenesis (Drost and Clevers, 2018), cell development (Betjes et al., 2021) and physiology. Standard cell-based assays such as fluorescence-activated cell sorting (FACS), quantitative real-time reverse transcription PCR (qRT-PCR) and single-cell RNA sequencing (scRNAseq) can be adapted for use with organoid cultures to identify baseline differences in inflammatory gene expression; assess reaction to inflammatory cytokines, other mediators or pathogens; and enable drug testing (Constant et al., 2022). Kim et al. (2020) and Tang et al. (2022) published detailed reviews on the development of organoid cultures and their applications in clinical and translational research.

### Development and validation of gastrointestinal bat organoids

**Fig. 1.**
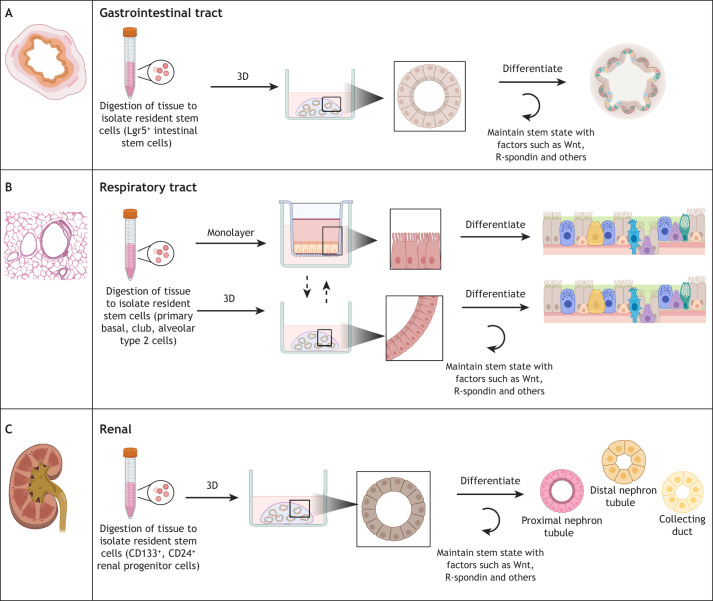
**Establishment of bat organoid cultures from gastrointestinal tract, respiratory tract and renal tissues.** Organoid cultures can be established from fresh or cryopreserved tissue samples by harvesting stem cells via enzymatic digestion or EDTA treatment. (1) Gastrointestinal organoids are cultured from intestinal Lgr5^+^ stem cells (Sato et al., 2009). (2) Respiratory tract organoids are cultured from respiratory stem cells including basal cells, club cells, and type 2 alveolar cells (Kellner et al., 2025). (3) Renal organoids are cultured from CD133^+^/CD24^+^ renal stem cells (Huang et al., 2021). The organoids can subsequently be maintained either as two-dimensional (2D) monolayers or as three-dimensional (3D) organoids embedded in an extracellular matrix, such as Corning Matrigel^®^ (Gunther et al., 2022; Kellner et al., 2025; Tang et al., 2022) in the presence of growth factors (typically a Wnt ligand, noggin and R-spondin), which support stem cell expansion and replication (Gunther et al., 2022). Other factors can be added to the media, such as ROCK inhibitors and TGF-β inhibitors, to prevent differentiation. Respiratory 3D organoids can be established from air–liquid interface primary cell monolayers (Chan et al., 2023). Alternatively, 2D monolayers of all three organoid types can be generated from 3D organoids (Chan et al., 2023; Kellner et al., 2025). Monolayer and 3D organoid cultures can be maintained under stem cell-promoting conditions for expansion or induced to differentiate by withdrawal of Wnt and supplementation with tissue-specific factors and media supplements, resulting in the development of multiple cell types (Urbischek et al., 2019; Yang et al., 2025). Lgr5, leucine-rich repeat-containing G-protein coupled receptor 5; TGF-β, transforming growth factor beta. Created in BioRender by Madden, S. R. (2026). https://BioRender.com/fouj7or.

**Fig. 2.**
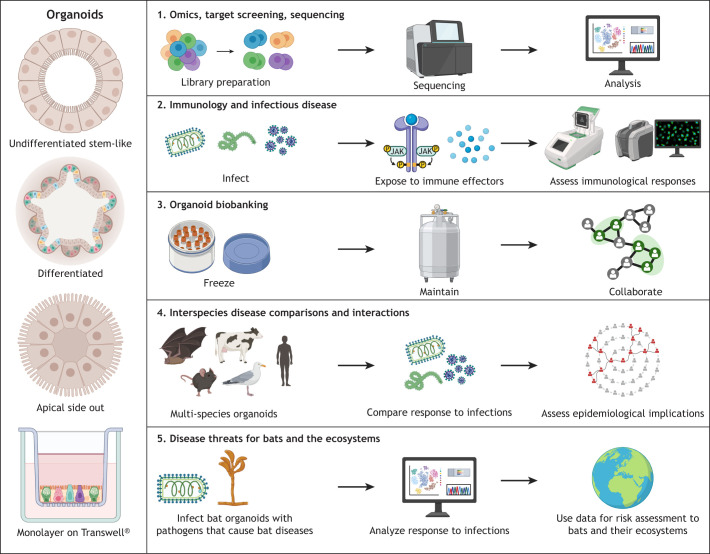
**Current and potential future research applications for bat organoids.** (1) For bat species with annotated genomes, organoids can be analyzed using transcriptomics or proteomics. Owing to the ability of organoids to retain the transcriptional signatures of their tissues of origin, gene expression analyses can be used to characterize baseline cellular functions and responses to various treatments (Hashimi et al., 2023; Kellner et al., 2025). (2) Organoids also enable study of immune and infection dynamics by exposing organoid cultures to pathogens and monitoring host immune responses (Hashimi et al., 2023). Exposure to immune effectors can be used to investigate alterations in homeostatic immunity (Chan et al., 2023; Kellner et al., 2025). (3) The development of bat organoid biobanks can further support reproducibility, facilitate scientific collaboration and maximize research output from bat tissues, which are a limited resource (Kim et al., 2025). (4) Comparative analyses of organoids derived from bats and other species, such as rodents or livestock, may be useful in the development of models for studying interspecies transmission. (5) Bat organoid model systems could, potentially, be used for studying infectious agents and environmental factors that affect or threaten bats in their natural habitats. JAK, janus kinase; P, phosphate group. Created in BioRender by Madden, S. R. (2026). https://BioRender.com/ih20ya6.

**Fig. 3.**
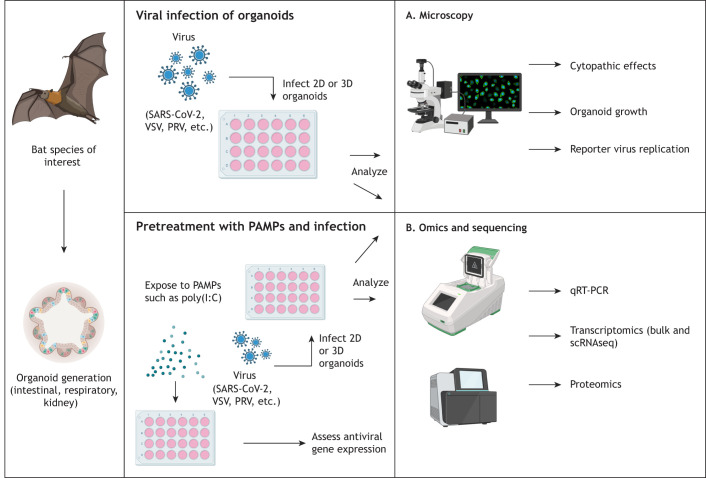
**Typical experiments for immunological analysis of viral infection.** Bat organoids derived from species of interest can be established, expanded, stimulated and/or experimentally infected with various viruses such as SARS-CoV-2, VSV and PRV. Pathogen-induced cytopathic effects or reporter virus replication can be assessed by microscopy, whereas immune responses, pathogen loads and gene expression can be quantified using qRT-PCR, RNA sequencing or proteomics (Hashimi et al., 2023; Kellner et al., 2025; Kim et al., 2025). To evaluate the effector functions of antiviral compounds, organoids can be pretreated with cytokines or other immune modulators, including PAMPs such as poly(I:C) (Liu et al., 2022). PAMPs, pathogen-associated molecular patterns; PRV, pteropine orthoreovirus; qRT-PCR, quantitative real-time reverse transcription PCR; SARS-CoV-2, severe acute respiratory syndrome coronavirus 2; scRNAseq, single-cell RNA sequencing; VSV, vesicular stomatitis virus. Created in BioRender by Madden, S. R. (2026). https://BioRender.com/z438xix.

The gastrointestinal tract is a major site of viral infection and shedding in bats (Hardmeier et al., 2021). Conversely, primary epithelial cell cultures from the intestine are difficult to maintain, and no transformed cell lines of the bat gastrointestinal tract have been developed. Therefore, the establishment and validation of bat gastrointestinal organoids has been a major advancement for the field. In the past few years, several research groups have devoted significant resources to creating, optimizing and characterizing organoid cultures from different bats and organ systems (Fig. 1). These organoid models have then been leveraged to study viral infection dynamics and cellular responses to viral infection (Figs 2 and 3). Since the publication of the first paper on bat organoids by Zhou et al. (2020), intestinal epithelial, airway epithelial, kidney and placenta organoid models have been successfully generated from various bat species, as summarized in Table 1. Bat organoids showed continuous growth for at least 6 months (Kim et al., 2025) and could be successfully re-grown after frozen storage (Hashimi et al., 2023). As described in more detail below, validation of organoid lines to confirm physiological cell composition and maintenance of tissue-specific gene expression patterns was performed using microscopy, proteomics and gene expression analysis.

**Table 1.** Overview of bat organoid lines that have been reported in the literature

Species	Common name	Organ	Viruses tested	Source
*Artibeus jamaicensis*	Jamaican fruit bat	Intestinal and gastric epithelium, placenta	SARS-CoV-2	Hashimi et al., 2023; Caldwell et al., 2025
*Carollia perspicillata*	Seba's short-tailed bat	Airway, lung	Swine IAV	Su et al., 2023
*Eonycteris spelaea*	Cave nectar bat	Trachea	PRV 3M	Chan et al., 2023
*Eptesicus serotinus*	Serotine bat	Trachea, bronchi, alveoli, kidney, intestinal epithelium	IAV (H1N1, H5N1, H9N2) orthohantavirus, MERS-CoV, SARS-CoV-2	Kim et al., 2025
*Hypsugo alaschanicus*	Alashanian pipistrelle bat	Trachea, bronchi, alveoli, kidney, intestinal epithelium	IAV (H1N1, H5N1, H9N2) orthohantavirus, MERS-CoV, SARS-CoV-2	Kim et al., 2025
*Myotis aurascens*	Steppe whiskered bat	Trachea, bronchi, alveoli, kidney, intestinal epithelium	IAV (H1N1, H5N1, H9N2) orthohantavirus, MERS-CoV, SARS-CoV-2	Kim et al., 2025
*Pipistrellus abramus*	Japanese house bat	Trachea, bronchi, alveoli, kidney, intestinal epithelium	IAV (H1N1, H5N1, H9N2) orthohantavirus, MERS-CoV, SARS-CoV-2	Kim et al., 2025
*Rhinolophus ferrumequinum*	Greater horseshoe bat	Trachea, bronchi, alveoli, kidney, intestinal epithelium	IAV (H1N1, H5N1, H9N2) orthohantavirus, MERS-CoV, SARS-CoV-2	Kim et al., 2025
*Rhinolophus sinicus*	Chinese rufous horseshoe bat	Intestinal epithelium	SARS-CoV-2	Zhou et al., 2020; Liu et al., 2022
*Rousettus aegyptiacus*	Egyptian fruit bat	Intestinal epithelium, nasal, tracheal, and bronchial epithelium	MARV, IAV H1N1, MERS-CoV, VSV	Kellner et al., 2025
*Rousettus leschenaultii*	Leschenault's rousette bat	Intestinal epithelium, lung	SARS-CoV-2, PRV	Elbadawy et al., 2021, 2025

IAV, influenza A virus; MARV, Marburg virus; MERS-CoV, Middle East respiratory syndrome coronavirus; PRV, pteropine orthoreovirus; SARS-CoV-2, severe acute respiratory syndrome coronavirus 2; VSV, vesicular stomatitis virus.

Horseshoe bats are a populous group of insectivorous bats that appear to be especially susceptible to coronaviruses (Lau et al., 2017; Lau et al., 2019; Zhou et al., 2020). Horseshoe bats were found to carry viruses with high sequence similarity to SARS-CoV-2 precursor viruses (Mallapaty, 2021; Zhou et al., 2021), which motivated the development of the first bat organoid model in 2020 (Zhou et al., 2020). Intestinal epithelial bat organoids were created from adult Chinese horseshoe bat (*Rhinolophus sinicus*) tissue by isolating intestinal crypts and using growth conditions similar to those for human and mouse intestinal epithelial organoids (Zhou et al., 2020). These organoids were cultured in Matrigel^®^ droplets with media that contained the recombinant murine growth factors Wnt, which promotes stem cell proliferation and maintenance; R-spondin, which stabilizes the Wnt receptor complex; and noggin, which prevents cell differentiation by inhibiting bone morphogenetic protein activation (Miyoshi and Stappenbeck, 2013) (Table 2). Importantly, withdrawal of Wnt induced epithelial cell differentiation, as expected, with morphologically distinct intestinal epithelial cell types – including enterocytes, goblet cells, Paneth cells and enteroendocrine cells – detected upon culture of the organoids in Wnt-free differentiation medium (Zhou et al., 2020). This study was an important proof of concept that, for at least one bat species, organoids could be developed with tools and protocols that work for other mammalian species. However, the Chinese horseshoe bat organoids in this study could only be maintained for four to five passages (Zhou et al., 2020).

**Table 2.** Media composition for bat organoid cultures

	Elbadawy et al. (2021)	Elbadawy et al. (2025)	Hashimi et al. (2023)	Kellner et al. (2025)	Kim et al. (2025)	Liu et al. (2022)	Su et al. (2023)	Zhou et al. (2020)
A83-01/SB-431542	−	+	+	+	+	+	+	+
B27	−	−	+	+	+	+	+	+
CHIR99021	−	−	−	−	+	−	−	−
EGF	+	+	+	+	+	+	+	+
FBS	−	−	−	+	−	−	−	−
FGF	−	+	−	+	+	−	−	−
Gastrin	−	−	+	−	−	+	−	+
Glutamax	−	+	−	+	+	+	−	+
HEPES	−	+	−	+	+	+	−	+
L-WRN	+	+	+	+	+	+	+	+
N-2	−	−	+	+	−	−	−	−
N-acetylcysteine	−	+	−	+	+	+	−	+
Nicotinamide	−	+	−	−	+	−	−	+
Penicillin/streptomycin	−	+	−	−	+	+	−	+
Primocin	−	+	−	+	+	+	−	+
SB 202190	−	−	−	−	+	+	+	+
TGF-α	+	+	−	−	−	−	−	−
Y-27632	−	−	+	+	+	+	−	+

A83-01/SB-431542, TGF-β inhibitor; blocks epithelial-to-mesenchymal transition; B27, cell growth support supplement, CHIR99021, synthetic Wnt activator; EGF, epidermal growth factor; FBS, fetal bovine serum; FGF, fibroblast growth factor; Glutamax, stable dipeptide form of L-glutamine; HEPES, 4-(2-hydroxyethyl)-1-piperazine-ethanesulfonic acid; L-WRN, supernatant from L-cells transformed with expression plasmids for murine Wnt, noggin and R-spondin; N-2, a cell growth supplement; N-acetylcysteine, antioxidant; Primocin, broad-spectrum antibiotic; SB-202190, a MAPK inhibitor; TGF-α: transforming growth factor alpha; Y-27632, ROCK pathway inhibitor.

Bats of the *Rousettus* genus are important reservoir hosts for human pathogens such as MARV (which causes severe hemorrhagic fever) and novel orthoreovirus (which causes human respiratory disease), making the development of culture protocols for organoids derived from these bats a priority (Amman et al., 2012; Hu et al., 2014). Elbadawy et al. (2021) were the first to establish organoids from Leschenault's rousette (*Rousettus leschenaultii*) intestine. Screening of different growth factors and media supplements showed that Wnt-3a, noggin, R-spondin, epidermal growth factor (EGF) and transforming growth factor alpha (TGF-α) were sufficient for the continuous growth of *Rousettus* intestinal organoids in culture (Table 2). Intestinal epithelial cell type differentiation in these organoids was confirmed using transmission electron microscopy (TEM). In addition, immunofluorescence staining with cross-reactive antibodies showed expression of intestinal epithelial markers, including E-cadherin, mucin (MUC)2 and leucine-rich repeat-containing G-protein coupled receptor 5 (Lgr5) (Elbadawy et al., 2021)*.* Importantly, Leschenault's rousette bat organoids could be successfully cultured for at least 10 months, which is comparable to reported culture periods for human (Sebrell et al., 2018) and murine (Miyoshi and Stappenbeck, 2013) organoids.

Similarly, using optimized media and culture techniques, the Liu group established Chinese horseshoe bat intestinal epithelial organoids that could be maintained in culture for at least 3 months (Liu et al., 2022). As in previous studies, intestinal epithelial cell differentiation was confirmed using TEM and immunofluorescence. In addition, this group used gene expression analysis to characterize the expression of genes involved in antiviral immunity at homeostasis. High baseline expression of interferons (IFNs) and interferon-stimulated genes (ISGs) has been previously reported in samples from bats and bat-derived cell lines (Clayton and Munir, 2020). To confirm these observations, IFN and ISG expression were assessed by using rapid amplification of cDNA ends (RACE) PCR and sequence data from the GenBank BioProject database. Compared to human intestinal epithelial organoids, the Chinese horseshoe bat organoids indeed expressed higher baseline levels of some ISGs (in particular 2′-5′ oligoadenylate synthase (*OAS1*) and 2′-5′ oligoadenylate synthase like (*OASL*) antiviral genes] and 1-3 log units higher baseline levels of *IFNA*, *IFNB*, *IFNG* and *IFNL1* and *IFNL3* (Liu et al., 2022). *IFNL* has previously been implicated in murine intestinal antiviral activity (Mordstein et al., 2010), and the elevated levels in Chinese horseshoe bat intestine organoids are indicative of strong antiviral potential. Therefore, results from these organoids models support the hypothesis that elevated baseline IFN expression is a common feature of bat immunity.

Jamaican fruit bats (*A. jamaicensis*) are New World bats commonly found in the Americas that are susceptible to different zoonotic pathogens, such as flaviviruses and coronaviruses (Machain-Williams et al., 2013; Munster et al., 2016). Hashimi et al. (2023) developed gastric and intestinal epithelial organoids from Jamaican fruit bats using standard methodology for intestinal crypt isolation and media with L-WRN supernatant containing murine Wnt, R-spondin and noggin, as detailed in Table 2. The organoids expressed microvilli and tight junctions, which are characteristic features of mature gastrointestinal epithelia, and contained mucus-producing cells. To assess correct representation of organ-specific gene expression patterns, gene expression of intestinal and gastric markers was confirmed using qRT-PCR. In contrast to previous work that found increased paracellular transport in bat intestinal epithelia (Brun et al., 2014), this study demonstrated that intestinal organoids established strong epithelial barriers when converted into monolayers, recapitulating a key physiological function of the mammalian gut (Hashimi et al., 2023). This group also expanded the bat organoid research toolkit by using mass spectrometry data with protein assignment based on an annotated bat genome to study epithelial differentiation and antiviral responses at the protein level (Hashimi et al., 2023). Overall, the development of gastrointestinal organoid lines has, for the first time, enabled studies of bat intestinal epithelial cells *in vitro*. Organoids now enable researchers to perform functional analyses of viral infection and antiviral responses in relevant primary cells and in multiple bat species.

### Development and validation of bat airway organoids

Chan et al. (2023) were the first to generate airway organoids from an Old World fruit bat, the cave nectar bat (*Eonycteris spelaea*), which is known to harbor a variety of zoonotic viruses, such as coronaviruses, pteropine orthoreoviruses (PRVs) and astroviruses (Chan et al., 2023). Cave nectar bats are easy to keep in captivity owing to their size and fast reproduction cycle, which makes them a convenient model organism. Chan et al. (2023) established a protocol for culturing primary airway epithelial cells from trachea tissue of cave nectar bats using air–liquid interface (ALI) culture in Transwells^®^, and then generated 3D airway organoids in Matrigel^®^, which could be maintained in culture for at least 6 weeks (Chan et al., 2023) (Fig. 2). Using the reference genome established by the same group (Wen et al., 2018), Chan et al. (2023) performed gene expression analysis and showed that both 3D airway organoids and monolayer ALI cultures were composed of forkhead box J1 (*FOXJ1*)*^+^* ciliated cells and tumor protein 63 (*TP63*)^+^ basal (stem) cells, which represent two key cell types in the tracheal epithelium. Addition of the cytokine interleukin (IL)-13 induced differentiation of a third, functionally important, tracheal cell type: mucus-producing, *MUC5AC*^+^ goblet cells. These validated bat respiratory tract organoids provide new opportunities for mechanistic follow-up experiments of results obtained *in vivo*, which will help replace and reduce animal experiments.

Building on the work described above, Su et al. (2023) developed airway organoids from Seba's short-tailed bats (*Carollia perspicillata*) to study the susceptibility of these bats to influenza A virus (IAV) and the bats’ potential role in zoonotic IAV transmission*.* Seba's short-tailed bats are susceptible to respiratory infection with H18N11 IAV (Gorka et al., 2020), one of only two bat-specific IAV variants that have been identified to date, which is, therefore, of great interest for understanding IAV ecology and its reassortment potential (Wu et al., 2014). Organoids from the conducting airways, i.e. the trachea and bronchi, were established by expanding adult tissue-derived stem cells and were maintained in a complex growth medium detailed in Table 2. The conducting airway organoids formed a characteristic pseudostratified epithelium with ciliated cells, goblet cells, club cells and basal cells. The conducting airway organoids also developed a functional epithelial barrier, as confirmed by measurements of transepithelial electrical resistance.

As mentioned above, bats of the *Rousettus* genus are a particularly relevant model system, as they harbor several important zoonotic viruses, such as MARV detected in the Egyptian fruit bat (*R. aegyptiacus*) (Amman et al., 2012). Kellner et al. (2025) used tissue samples from Egyptian fruit bats to generate airway organoids from the alveoli, the nasal, tracheal and bronchial mucosa, and the small intestine. scRNAseq was used to confirm that the organoids successfully modeled their respective tissues of origin by comparing tissue-specific gene expression signatures across the different organoids with native tissue samples (Kellner et al., 2025). Kellner et al. (2025) investigated baseline expression of immune genes in the absence of infection in Egyptian fruit bat airway organoids compared to human airway organoids. Similar to intestinal epithelial organoids derived from Chinese horseshoe bat tissues by Liu et al., 2022, the bat airway organoids showed higher baseline expression of both classical and alternative complement pathway genes, such as *CFH* and *C3*. Interestingly, *IFNE*, a type I IFN that is a known regulator of immunity and an antiviral effector, was also expressed at higher levels in Egyptian fruit bat small intestine organoids than in human small intestine organoids at baseline, again suggesting a higher level of baseline antiviral protection in bats than in humans (Kellner et al., 2025). However, in contrast to intestinal epithelial organoids from Chinese horseshoe bats (Liu et al., 2022), the expression of other IFNs in these organoids was not elevated compared to that in human organoids (Kellner et al., 2025). The inconsistent baseline IFN expression across bat species may be due to species-specific mechanisms of antiviral tolerance in bats and should be investigated in future studies to understand convergent evolution.

Elbadawy et al. (2021, 2025) continued their work with Leschenault's rousette bats (*R. leschenaultii*) and established bat lung organoids as a tool to study viral infection and tolerance to respiratory viruses. The group confirmed that cellular diversity in the lung organoids was largely consistent with that of native bat lung tissues and included multiple respiratory epithelial cell types such as secretory cells and basal cells. However, organoids lacked club cells, indicating that further refinement of culture conditions is required to maintain these crucial surfactant-producing cells (Elbadawy et al., 2025). The bat lung organoids also contained both alveolar airway type (AT)2 cells and airway basal cells. Because AT2 cells play an important role in lung homeostasis and can be transformed to AT1 cells that are necessary for air exchange, the group used FACS isolation to purify these cells. This approach enabled the generation of cystic luminal organoids with more flattened morphologies reminiscent of alveoli (Elbadawy et al., 2025). Similar alveolar organoids were successfully generated by Kellner et al. (2025) for *R. aegyptiacus.* Overall, these studies demonstrate that organoids can be generated from the respiratory epithelium of bats and can faithfully replicate typical characteristics of specific regions of the respiratory tract, including alveoli. Moreover, these studies have confirmed results obtained from other model systems showing increased baseline gene expression of antiviral IFNs in many bat species compared to humans.

### Other bat organoid models

In more recent studies, bat organoids have been generated from additional organ systems. Kim et al. (2025) established an extensive biobank of gastrointestinal, respiratory and urinary tract organoids from five species of insectivorous bats, four in the Vespertilionidae family and one in the Rhinolophidae family (Table 1). Importantly, this study was the first to generate organoids from bat kidneys, which are an important model for studying viral replication, given that many bat viruses are shed via the urine (Kim et al., 2025; McKee et al., 2022; Peel et al., 2019). All organoids were derived from wild-caught bats and could be maintained over 6 months in the culture conditions detailed in Table 2 (Kim et al., 2025). As in other studies, multiplex immunohistochemistry, TEM and scRNAseq of representative organoid types were used to confirm tissue-specific cell differentiation and composition. Cellular markers of kidney organoids included markers of monociliated tubular and ductal epithelial cells, proximal tubules, distal tubules, collecting ducts and proliferating progenitor cells, indicating that multiple nephron segments were represented in the cultures (Kim et al., 2025) (Fig. 1).

Expanding the bat organoid toolkit, a study by Caldwell et al. (2025) describes the generation of organoids from the trophoblast and decidua to model placental biology in Jamaican fruit bats. Overall, the development and characterization of multi-tissue, multi-species organoids have provided valuable biological tools for investigating bat immunology and host–virus interactions at intestinal, respiratory and kidney epithelial barriers. Studies using bat organoids have also confirmed findings from *in vivo* studies and other tissue culture models that point to a high baseline expression of antiviral genes in bats as a potential mechanism for enhanced antiviral mechanisms in bats compared to other mammals.

## Viral infection and host responses in bat organoids

### Susceptibility of bat organoids to viral infection

The main rationale for developing bat organoids was to generate physiologically relevant *in vitro* models for investigating susceptibility of different bat epithelial cell types to viral infection and host antiviral responses in bats. Several studies assessed cellular susceptibility based on viral receptor expression prior to performing infection experiments, typically using immunofluorescence staining, qRT-PCR or scRNAseq. For example, intestinal and lung organoids derived from Leschenault's rousette bats expressed angiotensin-converting enzyme 2 (ACE2) and transmembrane protease serine (TMPRSS)2 proteins, required for SARS-CoV-2 infection (Elbadawy et al., 2021, 2025). Likewise, intestinal organoids derived from Jamaican fruit bats by Hashimi et al. (2023) expressed low levels of ACE2. Consistent with expression patterns in tissue, lung and tracheal organoids derived from Seba's short-tailed bat (*C. perspicillata*) by Su et al. (2023) contained large amounts of α2,3-linked sialic acid, which is required for the cellular entry of many avian IAVs (Rather et al., 2025). Kellner et al. (2025) queried scRNAseq datasets from Egyptian fruit bat nasal, bronchial, alveolar and small intestinal organoids to identify expression of entry factors for several zoonotic viruses. They identified expression of Niemann-Pick C1 (*NCP1*), encoding the receptor for Ebola viruses and MARV; *ACE2*, encoding the entry factor for SARS-CoV-1 and SARS-CoV-2; ephrin B2 (*EFNB2*), encoding the receptor for Hendra and Nipah viruses; and *TMPRSS2* and *TMPRSS4*, which encode entry factors for multiple human pathogens (Kellner et al., 2025).

The coronavirus disease 2019 (COVID-19) pandemic motivated research with bat organoid models to study SARS-CoV-2. Zhou et al. (2020) were the first to infect bat organoids with SARS-CoV-2, using intestinal organoids derived from Chinese horseshoe bats. The group showed that levels of viral genome copies following infection of bat and human organoids were equivalent, pointing to a similar capacity of bat and human cells to support SARS-CoV-2 replication, consistent with the hypothesis that this virus may have originated from horseshoe bats (Zhou et al., 2020). In a follow-up study, Liu et al. (2022) confirmed that intestinal organoids derived from Chinese horseshoe bats supported SARS-CoV-2 infection but required a higher concentration of infectious virus than human intestinal organoids for viral replication, possibly due to the adaptation of the virus to human cells. Similarly, Kim et al. (2025) detected robust replication of SARS-CoV-2 in intestinal, but not respiratory tract, organoids from greater horseshoe bats (*Rhinolophus ferrumequinum*). Airway and intestinal organoids from *E. serotinus*, *Hypsugo alaschanicus*, *Myotis aurascens* and *Pipistrellus abramus* also did not support SARS-CoV-2 replication (Kim et al. (2025)*.* Leschenault's rousette bat intestinal and airway organoids expressed entry factors, including ACE-2, required for SARS-CoV-2 infection, but there was no evidence of viral entry or replication in either organoid type (Elbadawy et al., 2021). Likewise, Chan et al. (2023) were unable to show substantial replication of Wuhan-Hu-1 and omicron strains of SARS-CoV-2 in airway organoids from cave nectar bats. Hashimi et al. (2023) analyzed viral RNA in the cells, culture supernatant and subgenomic viral RNA of Jamaican fruit bat intestinal organoids infected with SARS-CoV-2 (USA-WA1/2020) and observed concentration-dependent increase in viral nucleic acids, but no significant release of infectious virions, pointing to incomplete or inefficient replication. Importantly, these data are consistent with results from *in vivo* infection experiments in Jamaican fruit bats, which showed no significant SARS-CoV-2 shedding beyond day 2 post-infection (Burke et al., 2023). Overall, these studies suggest that horseshoe bats are particularly susceptible to SARS-CoV-2-like coronaviruses and that the intestinal epithelium is a preferred site of SARS-CoV-2 replication.

By contrast, *in vivo* infection of Jamaican fruit bats with the related β-coronavirus, MERS-CoV, resulted in robust viral shedding from the respiratory and intestinal tract, although none of the bats developed clinical disease (Munster et al., 2016). MERS-CoV also replicated robustly in organoids derived from multiple other bat species, including greater horseshoe bats (*R. ferrumequinum*), Steppe whiskered bats (*M. aurascens*) and serotine bats (*E. serotinus*), but not in those derived from Japanese house bats (*P. abramus*) and Alashanian pipistrelle bats (*H. alaschanicus*) (Kim et al., 2025). These data indicate that although bat cells can tolerate many different viruses, they are not indiscriminately susceptible to all viruses, even when appropriate viral entry receptors are expressed.

IAVs have a strong zoonotic potential and can infect many animal species, including poultry, pigs, horses and bats (Klivleyeva et al., 2025). Infection of airway organoids derived from Seba's short-tailed bat (*C. perspicillata*) with two strains of swine IAV (H1N1/2006 or H3N2/2007) showed that the bat airway epithelial cells were highly susceptible to swine IAVs that target α2,3-linked sialic acid (Su et al., 2023). Kim et al. (2025) infected airway organoids derived from multiple bat species and organ systems with different influenza strains and showed that most species supported replication of human H1N1, avian H5N1 and bat H9N2 IAV, but not influenza B infection (Kim et al., 2025). Given that bats are not generally thought to be an important reservoir species or reassortment vessel for IAVs that infect humans or agricultural animals, the ability of primary bat cells to support replication of multiple different IAVs underscores the need to consider bats as potential factors in IAV outbreaks.

Another virus with zoonotic potential is PRV, which mainly causes subclinical infections in flying foxes but can also be transmitted to humans, in whom it causes fevers, diarrhea and acute respiratory illnesses (reviewed by Tan et al., 2017). Elbadawy et al. (2021, 2025) investigated PRV infection in intestinal and lung organoids derived from Leschenault's rousette bat and found that intestinal organoids, but not lung organoids, supported PRV replication and exhibited a strong cytopathic effect. Conversely, Chan et al. (2023) investigated PRV infection in cave nectar bat airway epithelial monolayers cultured under ALI conditions and confirmed robust infection. As in the studies by Elbadawy et al. (2021, 2025), cytopathic effects, as evidenced by plaque formation, were also induced in the ALI cultures (Chan et al., 2023). These data confirm previous reports that indicated that orthoreoviruses have a broad host range and can directly cause host cell death as a mechanism of pathogenesis (Tan et al., 2017).

Kellner et al. (2025) used bat alveolar and intestinal organoids and nasal organoid-derived ALI monolayers derived from Egyptian fruit bat to investigate bats' susceptibility and antiviral response to MARV and compared these findings with those from infection of human bronchial organoid-derived epithelia differentiated at an ALI. The group confirmed that infection with MARV Musoke isolate was supported by all tested organoids, with the highest viral levels observed in bat small intestinal organoids (Kellner et al., 2025). These data support the use of organoid models for studying the role of bats in this important and lethal zoonotic infection and for further mechanistic investigations into the disparate disease outcomes in bats and humans.

To determine whether bats are susceptible exclusively to bat-specific viruses or can support a broader range of viruses, including human-specific pathogens, Liu et al. (2022) tested intestinal epithelial organoids derived from Chinese horseshoe bats for susceptibility to both a bat-specific coronavirus (CoV-HKU4) and a human-specific enterovirus (EV-71). Analysis of the dose that infects 50% of a tissue culture (TCID_50_) and viral genome copies revealed that Chinese horseshoe bat intestinal organoids supported replication of CoV-HKU4, but only limited replication of EV-71. Surprisingly, human organoids supported replication of both the human EV-71 and the bat CoV-HKU4 virus. Consistent with the findings described for SARS-CoV-2 above, these data confirm that although bats have evolved to tolerate infection with numerous viruses, bat cells do not indiscriminately allow any virus to infect and replicate.

Kim et al. (2025) utilized their bat organoid biobank as a tool to isolate previously uncharacterized bat viruses from bat fecal samples and to identify potential pathogenic viruses based on the induction of cytopathic effects. This approach led to the identification of a new mammalian orthoreovirus and a Shaanvirus-like paramyxovirus that were able to replicate in tracheal organoids derived from multiple bat species and induced antiviral responses. The group also demonstrated the ability of the common antiviral drug remdesivir to suppress replication of MERS-CoV and mammalian orthoreovirus in bat tracheal organoids (Kim et al., 2025).

Together, these infection studies have demonstrated the potential of bat organoid models for studying the susceptibility of specific bat species and tissues to certain viruses, for performing side-by-side comparisons of viral infection in bats and other animals, for the isolation of new viruses from bat samples, and for assessing virulence in species-specific model systems.

### Innate immune responses of bat organoids to viral infection

To understand how bats can carry many viruses without developing clinical disease, researchers have focused on bat-specific innate antiviral immune mechanisms. Liu et al. (2022) treated intestinal epithelial organoids derived from Chinese horseshoe bats and from humans with poly(I:C), a toll-like receptor (TLR)3 agonist, and assessed changes in ISG and IFN gene expression (Liu et al., 2022). Compared to human intestinal organoids, the innate immune response of the bat organoids was more robust, as determined by a more rapid and prolonged expression of type III IFNs (particularly *IFNL1*) and ISGs [including *ISG15*, interferon-induced GTP Binding protein (*MX1*) and melanoma differentiation associated gene 5 (*MDA5*)] (Liu et al., 2022). The group also found that poly (I:C)-treated bat organoids expressed the proinflammatory genes *IL6*, *TNF* and C-X-C motif chemokine (*CXCL10*), indicating that the bat intestine mounts both an anti-inflammatory and an antiviral response to viral nucleic acids (Liu et al., 2022). These findings were confirmed in another study, in which researchers exposed Jamaican fruit bat intestinal organoids to TLR2 and TLR3 agonists, which led to significant upregulation of both inflammatory and IFN gene expression 6 h after inoculation (Hashimi et al., 2023).

Chan et al. (2023) infected ALI-differentiated airway epithelial cell cultures derived from cave nectar bat airway organoids with PRV, which also triggered both an antiviral and an inflammatory response, as determined by qRT-PCR. Together with strong induction of *IFNA* and *IFNB* expression, viral pattern recognition receptors such as *RIG-I*, antiviral protease genes such as *MX1*, and pro-inflammatory chemokines such as *CXCL10* were increased at least tenfold at 24 h post infection. Kim et al. (2025) used their bat organoid biobank to evaluate antiviral responses in tracheal and bronchoalveolar bat organoids in response to either MERS-CoV or IAV. In general, exposure to these viruses led to an increase in ISG expression, but the magnitude of the response varied across different pathogens and different bat species (Kim et al., 2025). For example, ISG induction was stronger in organoids from greater horseshoe bats than in those from serotine bats. In tracheal organoids from greater horseshoe bats, ISG induction was more pronounced in response to IAV than to MERS-CoV (Kim et al., 2025). Infection of alveolar organoids derived from Egyptian fruit bats with MARV also led to a significant induction of ISGs, including *MX1*, as determined by RNA sequencing (Kellner et al., 2025). Interestingly, a particularly robust increase in the expression of *IFNL*-like genes was found in bat alveolar organoids upon MARV infection. By contrast, the ISG response of ALI-cultured human bronchial organoids to MARV was markedly lower (Kellner et al., 2025). Importantly, recombinant bat type III IFN (i.e. IFN-λ) induced a strong antiviral response in bat alveolar, nasal and intestinal organoids and also protected them from infection with other viruses such as vesicular stomatitis virus (Kellner et al., 2025). Overall, these studies confirm the ability of bat cells to respond to viral infection with both antiviral and inflammatory gene expression patterns and point to a role for type III IFN in bat mucosal immunity.

## Advantages and limitations of bat organoids

Since the publication of the first study on intestinal organoids derived from Chinese horseshoe bats in 2020, bat organoid research has grown exponentially, now (April 2026) representing at least 11 bat species and nine different tissues. With appropriate culture conditions, accurate representation of tissue specific cells was achieved, as shown by scRNAseq and other methods, rendering the organoid models highly physiologically relevant. In comparison to human cells, higher baseline expression of complement factors and type I and type III IFNs, including IFN-ε and IFN-λ, was observed in intestinal and respiratory organoids derived from multiple bat species (Kellner et al., 2025; Liu et al., 2022), consistent with *in vivo* findings in bats and other *in vitro* systems (Clayton and Munir, 2020). Bat organoids were susceptible to *in vitro* infection with several different viruses and could also be successfully used to isolate new viruses from field samples (Kim et al., 2025). Importantly, bat organoids enable laboratories that do not have access to live bats or that have limited funds to perform extended experimental work upon procuring an initial batch of bat tissue. Furthermore, biobanking of established bat organoid lines will enable further expansion of collaborative bat research.

However, bat organoid models also have some limitations. As a reductionist model, epithelial organoids may not always accurately replicate observations made in live bats, and, to date, no study has directly compared *in vivo* infection with organoid infection for any bat species or virus. One other limitation is that culture media to maintain organoids require high concentrations of growth factors that may impact cell function and antiviral responses. In addition, small-molecule inhibitors that are commonly added to organoid cultures such as TGF-β inhibitor or ROCK inhibitor affect many cellular pathways and, thus, experimental results. Furthermore, epithelial organoids derived from adult tissue-specific stem cells lack immune cells that impact epithelial responses. However, this limitation could be overcome with co-culture models (Nozaki et al., 2016; Sebrell et al., 2019). Finally, organoids may harbor persistent, yet unidentified, viruses, posing a potential infection risk (Calisher et al., 2006), especially if the organoids are generated from wild-caught bats rather than bats maintained in captive research colonies. Appropriate biosafety measures should be implemented in any primary cell culture work with bat tissues.

## Key questions and challenges

Overall, bat organoids have emerged as a useful tool with potential to provide new insights into species- and tissue-specific characteristics of antiviral responses in bats and to help understand the unique role of bats as reservoir hosts for zoonotic viruses (Figs 1-3). Among the antiviral pathways described in multiple studies with organoids and live bats, type III IFNs, i.e. IFN-λ, have emerged as key players. These mediators may be linked to the generally increased ability of bats to tolerate viruses, given the role of type III IFNs in protecting barrier tissues from viral challenges while helping to maintain tolerance (Broggi et al., 2020) and should therefore be analyzed in more detail. With organoids, comparative high-throughput experiments for multiple species under standardized conditions are now feasible, as shown by Kim et al. (2025). Further expansion of bat organoid biobanks is needed to perform such cross-species studies and to unravel whether observed antiviral responses are common to all bats and viruses, or specific to unique bat species and pathogens.

One challenge with current bat research is the lack of species-specific reagents including growth factors for organoid cultures and antibodies for cell characterization and identification. The lack of species-specific growth factors may be overcome by using culture systems with stromal cells as a source of tissue-specific mediators (Bertaux-Skeirik et al., 2016; Lin et al., 2023). Recent advances in transcriptomics, particularly scRNAseq, and data-independent acquisition proteomics approaches now enable unbiased screening of bat cells without the need for specific reagents. However, these approaches require well-annotated reference genomes, which also are not yet available for all relevant bat species. Developing high-quality reference genomes for bats should be prioritized, as it would enhance multiple areas of bat research along with organoid studies.
